# Innovative Optogenetic Strategies for Vision Restoration

**DOI:** 10.3389/fncel.2018.00316

**Published:** 2018-09-21

**Authors:** Cameron K. Baker, John G. Flannery

**Affiliations:** ^1^Department of Molecular and Cell Biology, University of California, Berkeley, Berkeley, CA, United States; ^2^School of Optometry, University of California, Berkeley, Berkeley, CA, United States

**Keywords:** optogenetics, vision restoration, retina, opsin, GPCR, retinal degeneration

## Abstract

The advent of optogenetics has ushered in a new era in neuroscience where spatiotemporal control of neurons is possible through light application. These tools used to study neural circuits can also be used therapeutically to restore vision. In order to recapitulate the broad spectral and light sensitivities along with high temporal sensitivity found in human vision, researchers have identified and developed new optogenetic tools. There are two major kinds of optogenetic effectors employed in vision restoration: ion channels and G-protein coupled receptors (GPCRs). Ion channel based optogenetic therapies require high intensity light that can be unsafe at lower wavelengths, so work has been done to expand and red-shift the excitation spectra of these channels. Light activatable GPCRs are much more sensitive to light than their ion channel counterparts but are slower kinetically in terms of both activation and inactivation. This review article examines the latest optogenetic ion channel and GPCR candidates for vision restoration based on light and temporal sensitivity.

## Introduction

**Figure 1:**
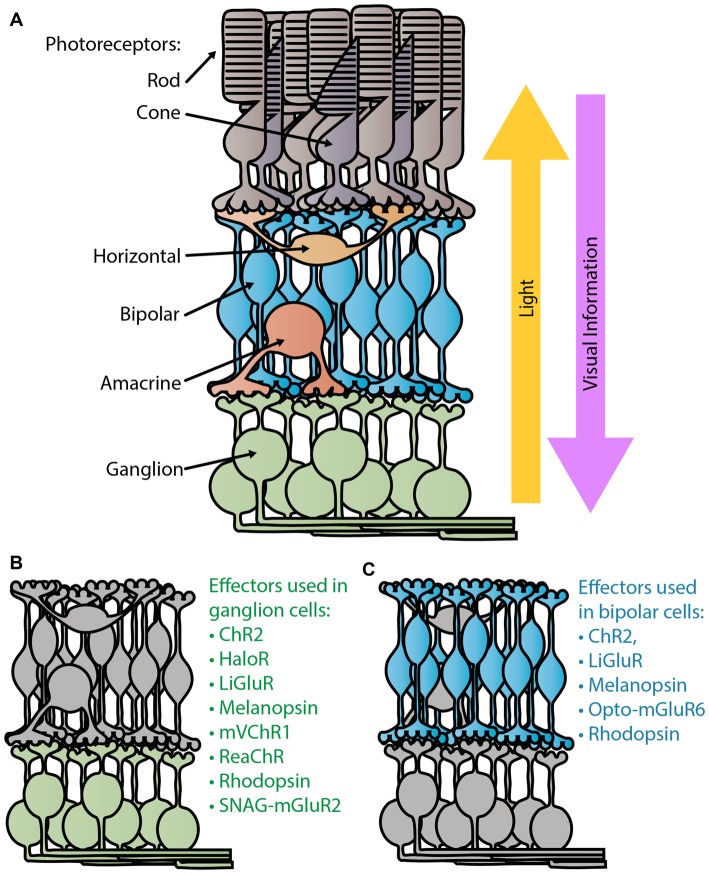
Retina schematic. **(A)** Diagram of a normal healthy retina. Light passes through the retina, entering through the retinal ganglion cell (RGC) layer to reach the light sensitive photoreceptors, the rods and cones, in the outer retina. Visual information is sent from the photoreceptors to the bipolar cells where the ON/OFF processing begins. Ganglion cells are the terminal retinal signal recipients and they relay onto neurons in the lateral geniculate nucleus in the thalamus. Panels **(B,C)** depict the degenerate retina without photoreceptors. Panel **(B)** lists the optogenetic therapies that have been tested in ganglion cells (Bi et al., 2006; Lin et al., 2008; Zhang et al., 2009; Caporale et al., 2011; Tomita et al., 2014; Sengupta et al., 2016; Berry et al., 2017), while **(C)** lists those tested in bipolar cells (Lagali et al., 2008; Gaub et al., 2014, 2015; Macé et al., 2015; Scalabrino et al., 2015; van Wyk et al., 2015).

Shortly after the popularization of optogenetic tools in neuroscience began in the early 2000s (Nagel et al., 2003, 2005; Boyden et al., 2005), researchers began evaluating their use in a novel *in vivo* application: vision restoration. In America, over 1,000,000 people are currently considered blind, with that number expected to double by 2030 (National Eye Institute, 2010). Most patients suffering from retinal degenerative diseases, like retinitis pigmentosa and macular degeneration, often first lose their light sensitive photoreceptors, the rods and cones, leaving the remaining retinal tissue light insensitive (Figure 1). However, the surviving cells can retain functionality and connections to the brain long after photosensitivity disappears. For decades researchers have attempted to activate this remaining tissue with prosthetic electrical stimulation (Margalit et al., 2002). With the advent of optogenetics, photosensitivity and vision can be restored at cellular resolution.

**Figure 2:**
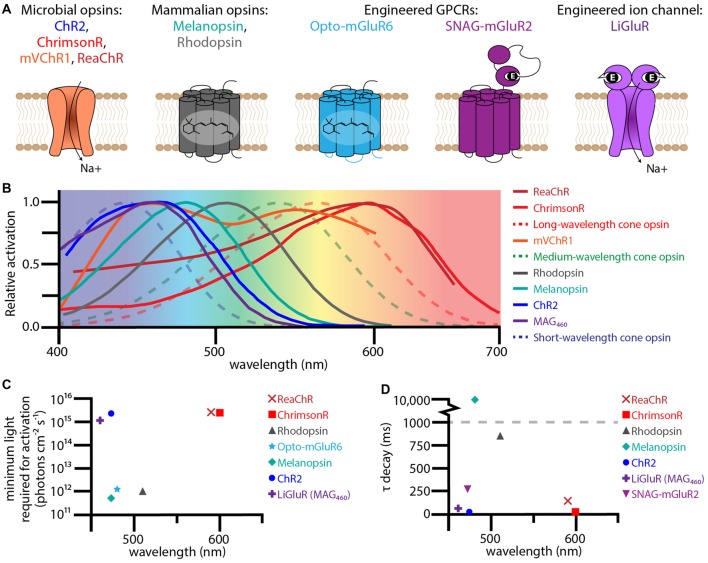
Therapeutic optogenetic effectors used to restore the visual response in degenerate retinas. **(A)** Structural diagrams of optogenetic microbial opsins, mammalian opsins, and engineered GPCRs and ion-channels. The microbial opsins are all sodium permeable ion channels. The mammalian opsins, melanopsin and rhodopsin, are GPCRs with six transmembrane domains containing the chromophore 11-cis retinal. The engineered GPCR Opto-metabotropic glutamate receptor 6 (mGluR6) is comprised of the transmembrane domains from melanopsin with the intracellular loops from mGluR6. SNAG-mGluR2 is mGluR2 with a N-terminal SNAP-tag that tethers the PORTL BGAG. Upon light stimulation, the azobenzene in BGAG isomerizes allowing the distal glutamate to bind to the active site of mGluR2. The engineered ion channel LiGluR is iGluR6 with a cysteine mutation that allows for the covalent binding of the photoswitch maleimide-azobenzene-glutamate (MAG). Light isomerizes the azobenzene in MAG forcing the glutamate into the binding pocket. **(B)** Excitation spectra for optogenetic effectors used for vision rescue (solid lines) and human cone opsins (dotted lines). **(C)** The minimum light required for activation for various optogenetic effectors when used for vision rescue plotted against wavelength. **(D)** The τ decay constant plotted against wavelength for various optogenetic effectors. The excitation spectra, minimum light requirements, and τ decay constants were collected from the following publications: Lin et al. (2008, 2009); Gaub et al. (2014, 2015); Tomita et al. (2014); van Wyk et al. (2015); Pruneau et al. (2016); Sengupta et al. (2016); Berry et al. (2017).

The first successful attempt at bestowing light sensitivity to non-photoreceptor retinal cells with optogenetic tools was in 2006 (Bi et al., 2006). In this pioneering work they heterologously expressed a microbial opsin, channelrhodopsin-2 (ChR2) from *Chlamydomonas reinhardtii* (Figure 2A), in thalamic projecting retinal ganglion cells (RGCs; Figure 1B) via adeno associated virus (AAV) serotype 2. This groundbreaking article showed that endowing surviving retinal cells with light sensitive proteins can restore light responses both retinally and cortically. Furthermore, it was one of the first articles to use AAV as a retina delivery vector and to demonstrate long term expression and safety. For over a decade researchers have been improving upon this basic method of virally expressing optogenetic proteins in surviving retinal cells, using new effectors to improve light and temporal sensitivity.

The ambitious aim to cure blindness optogenetically has driven light sensitive protein development, benefiting neuroscience as a whole with new effectors. Human vision has broad spectral (400–700 nm) and light sensitivity (10^4^ to 10^16^ photons cm^−2^ s^−1^) with high temporal resolution (up to 60 Hz for long cone opsins; Kalloniatis and Luu, 1995). In order to restore sight, researchers have had to step beyond ChR2 to find and engineer new optogenetics that can better recapitulate human vision. In this review article, we will compare the latest optogenetic ion-channel and G-protein coupled receptor (GPCR) based technologies in their effectiveness at restoring sight to blind retinas based on the amount of light required for activation and temporal sensitivity.

## Ion Channels

The main advantage of optogenetic ion channel based therapies is temporal sensitivity. Photoactivation allows ions to flow through the channel activating or inhibiting the neural host. Upon light stimulus removal, inactivation is quick. With an opening and closing rate on the order of milliseconds, optogenetic ion channels have the potential for successive high frequency stimulation required for normal human vision (Figure 2D). However, light sensitivity is sacrificed for temporal sensitivity, with most channels requiring at least 10^15^ photons cm^−2^ s^−1^ to activate their neural hosts (Figure 2C; Bi et al., 2006; Sengupta et al., 2016). This amount of light is dangerous at shorter wavelengths, like the 470 nm that maximally activates ChR2 (International Commission on Non-Ionizing Radiation Protection, 2013). Of growing interest is “red-shifting” these ion channels to safer long wavelengths.

One such ion channel that was successfully red-shifted is the modified mammalian ion channel LiGluR (Figure 2A; Volgraf et al., 2006). Based on the human ionotropic glutamate receptor 6, LiGluR has a mutated cysteine residue that allows Maleimide-Azobenzene-Glutamate (MAG), a photoswitchable tethered ligand (PTL), to covalently bind to the outside of the ion channel. Certain wavelengths of light isomerize the azobenzene from trans to cis, forcing the distal glutamate into the protein’s binding pocket opening the ion channel. The first generation of MAG, MAG0, was bistable and used 380 nm light to open and 500 nm to close. While this ultraviolet bistable channel was able to restore the visual response (Caporale et al., 2011), including the pupillary response, the amount and wavelengths of light required are dangerous to humans. To circumvent these problems, a second generation MAG, MAG460, was developed (Kienzler et al., 2013). No longer bistable and activated at 460 nm (Figure 2B), MAG460 was also able to restore the visual response in both mice and dogs (Gaub et al., 2014). While the LiGluR-MAG460 system requires a similar amount and spectrum of light as ChR2 (Figures 2C,D), its modular design is advantageous. Since LiGluR requires the PTL to be delivered in trans, patients would have the option of which one to have delivered and when, which would be especially beneficial if other PTLs with different activation spectrums are developed in the future.

Currently, in order to achieve activation using wavelengths above 550 nm, different opsins are required. In 2008 the Deisseroth group discovered the red-shifted ChR2 ortholog VChR1 from *Volvox carteri* (Zhang et al., 2008). This cation channel’s excitation spectrum is red shifted approximately 70 nm when compared to ChR2, with a peak excitation at 535 nm and a capacity to produce spiking at 589 nm. While this red-shifted spectrum was promising, this channel does not express well due to inefficient plasma membrane integration.

Various groups have tackled this expression problem by generating chimeras with other microbial ion channels. Of these chimeric microbial channels, ReaChR (Lin et al., 2013) and mVChR1(Tomita et al., 2014) have demonstrated vision restoration efficacy in rodents, and in the case of ReaChR, primates (Figures 1B, 2A; Sengupta et al., 2016). ReaChR, which used the N-terminus of ChiEF to facilitate membrane trafficking, the transmembrane domain from VChR2 to increase expression, and a L171I point mutation to reduce desensitization above 600 nm, has a red-shifted activation spectrum with a peak excitation ~600 nm and is capable of generating photocurrent at 630 nm (Figure 2B; Lin et al., 2013). In mice this channel could produce spiking frequencies up to 30 Hz in ganglion cells and 22 Hz in macaque retinal explants (Sengupta et al., 2016). Considering that film often uses 24 frames per second, the temporal sensitivity of ReaChR seems sufficient for vision rescue. While ReaChR still requires light on the same order of magnitude as ChR2 at 10^15^ photons cm^−2^ s^−1^ (Figure 2C), light at this portion of the spectrum is safe up to 10^17–18^ photons cm^−2^ s^−1^ (International Commission on Non-Ionizing Radiation Protection, 2013).

The other red-shifted chimeric channel, mVChR1, is a fusion of VChR1 and the N-terminus *Chlamydomonas* channelrhodopsin-1 (not to be confused with C1V1) and has the largest activation spectrum of the ion channel based opsins tested thus far, ranging from 468 nm to 640 nm (Figure 2B; Tomita et al., 2014). The conductance of mVChR1 is not as efficient as ChR2 and it also requires a similar amount of light to open the channel. However, it is the only ion channel responsive to light throughout the whole human visual spectrum, making it a promising gene therapy candidate. To further improve spectral sensitivity, the same group used both mVChR1 with ChR2 to restore vision in blind mice (Sato et al., 2017). Unsurprisingly, the use of multiple opsins generated greater light responses across the spectrum than one opsin alone.

In 2014 another red-shifted microbial ion channel was discovered in *Chlamydomonas noctigama*, termed ChrimsonR (Figure 2A; Klapoetke et al., 2014). With a similar activation spectrum and light requirements of ReaChR but with a faster deactivation time constant (Figures 2B–D), ChrimsonR is another strong vision restoration gene therapy candidate and is currently in clinical trials (ClinicalTrials.gov, 2015).

Even though the red-shifted opsin variants use a safer wavelength of light, they still require extremely bright light on the order of 10^15^ photons cm^−2^ s^−1^ (Figure 2C). Since the light is the direct effector of these ion channels, there is no way to increase light sensitivity of the system other than increasing the light sensitivity of the opsin itself. Currently, the best strategy to increase light sensitivity is to use a light sensitive effector with the capacity to amplify the light response, like GPCRs.

## GPCRs

While there have been great improvements in spectral sensitivity and conductance for light activated ion channels, they still pale in comparison to the signal generated by optogenetic GPCRs. The pay-off for this increased light sensitivity is a loss in temporal sensitivity, with optical GPCRs lagging behind their ion channel in terms of both activation and inactivation (Lin et al., 2008; Cehajic-Kapetanovic et al., 2015; Gaub et al., 2015; Berry et al., 2017; De Silva et al., 2017). However, the slower kinetics of the optogenetic GPCR could be well tolerated due the loss of a synaptic layer. Compared to wild type, the light signal was able to reach V1 faster for the optogenetic ion channel gene therapies by tens of milliseconds (Bi et al., 2006; Lagali et al., 2008; Caporale et al., 2011; Macé et al., 2015; Sengupta et al., 2016). The loss of the photoreceptors and their synapse means that there are fewer cells the light signal has to pass between before reaching the brain in these animals with restored vision. Due to this, the slowness of the GPCRs can be partially compensated for by signal generation in downstream cells.

The first GPCR to be adapted for vision restoration was the light sensor for intrinsically photosensitive RGCs, melanopsin (Figure 2A; Lin et al., 2008). Responsible for the pupillary light response and maintenance of circadian rhythms (Provencio et al., 1998), melanopsin is a clear candidate for vision restoration due to its established ability to generate light responses in non-photoreceptor retinal cells. When delivered intravitreally to the ganglion cell layer (Lin et al., 2008), or subretinally to outer retinal cells (De Silva et al., 2017), melanopsin treated retinas are three fold more light sensitive than any microbial opsin, only requiring 10^12^ photons cm^−2^ s^−1^ to generate a signal (Figure 2C). While melanopsin treated mice are able to perform some basic light response behavior and show an increased pupillary light response, the GPCR’s kinetics are incredibly slow. It takes hundreds of milliseconds to several seconds to activate melanopsin, and even longer for it to turn off (Figure 2D; De Silva et al., 2017). This slow response time is sufficient for basic perception (i.e., is it daytime or not), but makes it a poor tool for the high acuity vision associated with humans.

Another candidate for vision rescue is rhodopsin, the exceedingly light sensitive GPCR found in rod photoreceptors (Cehajic-Kapetanovic et al., 2015; Gaub et al., 2015). Rod photoreceptors are capable of responding to a single photon thanks to the phototransduction cascade. Light isomerizes the chromophore 11-*cis* retinal into all-*trans* retinal, inducing a conformational change in the GPCR which activates its G-protein, transducin. The α-subunit of transducin dissociates and activates phosphodiesterase (PDE), which lowers the concentration of cGMP, closing cyclic nucleotide gated channels and hyperpolarizing the cell. When expressed in non-photoreceptor cells, rhodopsin has a similar light sensitivity to melanopsin of 10^12^ photons cm^−2^ s^−1^ (Figure 2C), but importantly responds to light 10 times faster than melanopsin (Figure 2D; Cehajic-Kapetanovic et al., 2015; Gaub et al., 2015). However, when compared to rod photoreceptors, rhodopsin activation in ganglion cells or bipolar cells is much slower. When heterologously expressed, it is unlikely that the time to isomerize 11-*cis* or activate rhodopsin itself has changed, but rather the lack of other phototransduction cascade proteins increases cellular response times. Rod photoreceptors have specialized discs which contain all the phototransduction cascade proteins to promote efficient signaling. While other cells lack this specific structure and phototransduction cascade, photoactivation of other existing signaling cascades could improve temporal sensitivity.

What is impressive is that the rhodopsin protein is photosensitive at all. Many believed that rhodopsin outside a photoreceptor would be unable to attain its 11-*cis* retinal chromophore. 11-*cis* is tightly regulated being recycled from all-*trans* in retinal pigment epithelium (RPE) and Müller cells, which have developed specialized mechanisms to deliver the incredibly photosensitive pigment to photoreceptors (Kiser et al., 2014). While some teams have had to supply the 11-*cis* for the multielectrode array (MEA) experiments, *in vivo* assays demonstrated effective iterative activation of rhodopsin without the use of exogenous chromophore (Cehajic-Kapetanovic et al., 2015). Perhaps in the degenerate retina, the RPE and Müller cells still produce 11-*cis* and can aberrantly deliver it. Considering that photoreceptors contain approximately a thousand discs each with thousands of rhodopsin molecules (Nathans, 1992), the concentration of rhodopsin ectopically expressed in ganglion cells or bipolar cells would pale in comparison to wild type levels, perhaps low enough to ensure chromophore delivery despite being non-target cells. Furthermore, there might be alternative chromophore delivery mechanisms. It was recently determined that melanopsin also uses 11-*cis* (Walker et al., 2008), so whatever mechanism delivers the 11-*cis* to melanopsin could potentially also deliver it to rhodopsin when expressed in ganglion or bipolar cells.

Recently, optically controlled GPCRs have been engineered by multiple groups (Airan et al., 2009; Karunarathne et al., 2013; Levitz et al., 2013; Broichhagen et al., 2015; van Wyk et al., 2015; Morri et al., 2018). Unlike melanopsin or rhodopsin, these GPCRs have been constructed or modified to become light sensitive. One particularly interesting candidate, Opto- metabotropic glutamate receptor 6 (mGluR6), is a chimeric protein composed of the chromophore-adhering transmembrane domains of melanopsin with the regulatory transmembrane domains of mGluR6, the ON-bipolar specific GPCR (Figure 2A; van Wyk et al., 2015). Opto-mGluR6 is the most light sensitive construct tested so far, eliciting a light response at 5 × 10^11^ photons cm^−2^ at 473 nm (Figure 2C). In degenerate animals expressing Opto-mGluR6 in their bipolar cells (Figure 1C), the light responses generated signals in bipolar cells and ganglion cells had similar timing to photoreceptor evoked light responses in wild type animals. Expressing a photoactivable version of the naturally occurring GPCR in the target cell type is an enticing goal, however, the Opto-mGluR6 group had to resort to transgenic animals to show any function or behavior. In order for any of these therapies to be a viable option for people, non-transgenic routes must be pursued.

## Delivery Methods

The goal of these ambitious projects is to cure blindness in humans. While transgenic animals are an invaluable laboratory tool, they are not applicable to humans. Viral transduction is currently the best method to constitutively express heterologous proteins in mammals. Other methods like electroporation have been shown to successfully deliver transgenes in mice (de Melo and Blackshaw, 2011), but the AAV method has been demonstrated to be safe, effective and long lasting, as evidenced by the recent FDA approval for the first vision restoration gene therapy for LCA2 (Maguire et al., 2009; Simonelli et al., 2010; Jacobson et al., 2012; FDA, 2017).

The current trend in many laboratories is to try and recapitulate the ON/OFF light response by infecting ON-bipolar cells with improved viral capsids and promoters (Figure 1C; Doroudchi et al., 2011; Cronin et al., 2014; Gaub et al., 2014, 2015; Cehajic-Kapetanovic et al., 2015; Macé et al., 2015; van Wyk et al., 2015). The hypothesis is that infecting the furthest upstream cells preserves the circuitry and processing resulting in a better final signal sent to the brain. By using the mGluR6 promoter, the ON-bipolar cell specific metabotropic glutamate receptor, optogenetic gene expression is limited to these upstream cells. ChR2 (Doroudchi et al., 2011; Cronin et al., 2014; Macé et al., 2015), LiGluR (Gaub et al., 2014), rhodopsin (Cehajic-Kapetanovic et al., 2015; Gaub et al., 2015) and Opto-mGluR6 (van Wyk et al., 2015) mediated light activation of bipolar cells (Figure 1C) can produce ON and OFF responses in ganglion cells and diverse responses in V1, unlike the simple ON response produced by photosensitive ganglion cells.

While this is an admirable goal, bipolar cells are one of the hardest cell types to infect in the retina (Dalkara et al., 2009). Laboratories have engineered new and improved AAV variants with unprecedented retinal penetration (Dalkara et al., 2013), and even the best capsid variants do not efficiently transduce bipolar cells. AAV never achieves complete infection and is normally “patchy,” so complete restoration of a receptive field looks unlikely. Furthermore, the mGluR6 promoters used to restrict expression to the ON-bipolar cells may not work in many retinal dystrophies due to dis- and downregulation of mGluR6 (van Wyk et al., 2017). mGluR6 is tonically activated by the continuous release of glutamate by photoreceptors in the dark. Upon photoreceptor cell death, mGluR6 is no longer stimulated and the ON-bipolar cells undergo transcriptional changes that limit mGluR6 expression and potentially other genes under that promoter (Jones et al., 2016).

Instead of trying to restrict expression to bipolar cells, it might be more advantageous to use ubiquitous promoters that would allow expression in bipolar cells and other retinal cells to increase light sensitivity. This way some greater processing is preserved allowing for the generation of ON and OFF light responses, while generally increasing the light sensitivity of the retina as a whole.

## Multi-Effector Therapy

Bipolar cells direct the ON and OFF pathways in healthy tissue, but that does not mean that bipolar derived signal is the only way to generate ON and OFF signals. There are a variety of inhibitory optogenetic ion channels (Berndt et al., 2014, 2016; Govorunova et al., 2015), pumps (Schobert and Lanyi, 1982; Gradinaru et al., 2008; Chow et al., 2010) and GPCRs (Levitz et al., 2013; Broichhagen et al., 2015) that could emulate an “OFF” response. This was first demonstrated by Zhang et al. (2009) by combining the inhibitory ion pump HaloR with ChR2 to produce ON and OFF and ON/OFF light responses. While HaloR requires 20 times more light than ChR2, which already requires an unsafe amount of light, this study importantly shows that the retina can produce multiple types of light responses without bipolar cell transduction.

Recently, Berry et al. (2017) improved upon Zhang’s original work by using their engineered SNAG-mGluR2 and LiGluR combination therapy. Similar to LiGluR, SNAG-mGluR2 is a modified version of the mGluR2 with a N-terminal SNAP tag that allows stable conjugation of the azobenzene-glutamate photoswitch by a selective benzylguanine-reactive group (Figure 2A; Broichhagen et al., 2015). SNAG-mGluR2 is one of the fastest optogenetic GPCRs with kinetics on the order of hundreds of milliseconds (Figure 2D), but it unfortunately requires a similar amount of light as ChR2 (Figure 2C). The combination of the excitatory LiGluR ion channel and inhibitory SNAG-mGluR2 GPCR generates diverse light responses including ON, OFF and ON/OFF responses. These diverse responses improved visual behavior in treated mice compared to LiGluR or SNAG-mGluR2 alone. While this combination therapy still requires bright light, it importantly shows that multi-effector therapy has the potential to restore complex and diverse cellular light responses similar to natural vision.

## Conclusions

The audacious goal of genetically restoring vision to the blind is now possible. Newly discovered and developed optogenetics have improved upon the original ChR2 studies. By red-shifting ion channels, researchers have made safer alternatives with broader spectrums. And new GPCRs are gaining speed to allow for the temporal precision required for high acuity vision. The new trend to use multiple effectors to generate diverse responses should be expanded further. Humans use three different cone opsins and one rod opsin to generate vivid visual perception. With so many optogenetics with diverse excitation spectra, combination therapies using multiple effectors producing excitatory and inhibitory responses at different wavelengths could generate the complex visual information comparable to responses naturally derived in healthy tissue. With new and innovative therapies constantly being developed, vision restoration is within sight.

## Author Contributions

CB devised the review article. CB and JF wrote it.

## Conflict of Interest Statement

The authors declare that the research was conducted in the absence of any commercial or financial relationships that could be construed as a potential conflict of interest.
